# Renal Apoptosis in the Mycotoxicology of *Penicillium polonicum* and Ochratoxin A in Rats

**DOI:** 10.3390/life12030352

**Published:** 2022-02-28

**Authors:** Ana Miljkovic, Peter Mantle

**Affiliations:** Biochemistry Department, Imperial College London, London SW7 2AZ, UK; anabrake@gmail.com

**Keywords:** *Penicillium polonicum*, karyocytomegaly, apoptosis, confocal microscopy, propidium iodide, nephropathy, *Penicillium aurantiogriseum*, ochratoxin A, DNA adduct

## Abstract

*Penicillium polonicum* K. M. Zaleski, which is common on foodstuffs in Balkan regions that are notable for their history of endemic nephropathy, has been shown experimentally to cause a striking histopathological renal change in rats that are given feed contaminated by this fungus. The nephrotoxic agent(s) are only partially characterized. The principal change seen in the cortico-medullary region is karyocytomegaly, but apoptosis, identified with the ApopTag^®^ methodology, is the first response to a dietary extract of *P. polonicum*-molded wheat after a few days of exposure. Chromatin debris migrates along the nephrons into the medulla, but whether the damaged epithelial fate is via autophagy is unclear. In intermittent exposure experiments, renal apoptosis was resolved with the cessation of exposure and was restored with renewed exposure. Apoptosis became less evident after 3 months of chronic exposure. In contrast, a relatively high dose of dietary ochratoxin A, a potent nephrocarcinogen in male rats after many months of dietary exposure, gave no evidence of apoptosis in asymptomatic weanlings over a few days of dietary exposure. This was attributed to a masking effect by concomitant marked histological disruption in renal tissue. However, in young adults, renal apoptosis was a primary outcome of dietary exposure to either the *P. polonicum* extract or to ochratoxin A, but the histopathological response to the former was less distorted. The apparent conflicted use in the literature of *P. polonicum* as a descriptor is highlighted.

## 1. Introduction

The topic of renal apoptosis in response to fungus is contemporary with the history of mycotoxicology, founded partly by the discovery of ochratoxin A (OTA) [1] and its relevance to porcine renal disease [2]. It also encompasses the Balkan endemic nephropathy (BEN), which was first recognized in Bulgaria in the 1950s as a silent bilateral renal atrophy and manifested as the principal cause of human mortality in particular agricultural communities. These communities were usually in low-altitude (flood plain) geographical clusters, as well as in Romania and the former Yugoslavia. An early observation of the increase in deaths during those years experiencing high rainfall [3] prompted a forensic focus on molds. Despite the prevailing geopolitical limitations, a study in the hyperendemic Romanian village of Erghevita [4] enabled the sampling of mycobiota associated with human foodstuffs, from which a common green-sporing fungus, identified as *Penicillium verrucosum* var. *cyclopium* Westling (IMI 180992), formed the basis for subsequent experimental toxicology [5]. The principal findings were renal toxicity in rats when given as a dietary additive, and the specific growth inhibition of renal cells in tissue culture. The former was expressed as karyocytomegaly in nephron epithelia in the cortico-medullary region. Concurrently, since there was increasing recognition of an association of urothelial tumors with some cases of Balkan nephropathy, the karyomegaly in *Penicillium*-treated rats was seen as possibly a pre-tumor change. Meanwhile, taxonomic revision within the *Penicillium* subgenus *Penicillium* [6] revised *P. verrucosum var. cyclopium* to within *P. aurantiogriseum* Dierckx, in response to which further study focused on its nephrotoxicity and abundance in households in the Croatian village of Kaniza [7], notable as hyperendemic for BEN. The same fungus was common in an analogous cluster of villages in the Vratza region in NW Bulgaria [8]. Attempts to characterize the toxin(s) causing rat renal karyomegaly and its histopathology continued [9], while its failure to affect the hamster was also demonstrated [10]. Renewed experimental attention to compare rat nephropathies of *P. aurantiogriseum* with that of the mycotoxin OTA resumed in the 1990s. Apoptosis located amid karyomegalic changes then became possible with the ApopTag staining methodology and raised questions about any etiological application to the renal atrophy of BEN [11]. Unfortunately, merely a brief explanation and color illustration of this in situ apoptosis, revealed by the fluorescent TUNEL staining of 3′-OH caspase, now appears online only and without its color.

Further taxonomic revision of *P. aurantiogriseum* [12] defined four new species descriptions while retaining that name for a more restricted group of Penicillia. One of the new redefined species is *P. polonicum*; rat nephrotoxicity has seemed exclusive to this species and that descriptor has accordingly been applied ever since.

An exceptional opportunity for a comparison of rat renal histopathology, in response to an extract of *P*. *polonicum* fermentation, with that of a vervet monkey in South Africa found no change in the primate in contrast to the striking renal histopathological changes in the rat [13]. This at least implied no apparent human genotoxic risk from susceptibility to a *P. polonicum* karyocytomegaly, even when a primate model had been given an excessively large dose of extract relative to the rat model. However, the expression of karyomegaly in non-human primates has only been recorded infrequently [14] and, in general, a chemically induced karyomegalic response in the rat does not necessarily predict a similar change in human kidneys. Hard [14] also recommended that the threshold for diagnosing renal tubule karyomegaly in animal studies should be accepted as requiring at least four times that of normal nuclear size. This was not quite satisfied for one rat renal example attributed to *P. polonicum* [15], being just one of a small group of rat renal tumors caused experimentally by OTA and subjected to special DNA ploidy distribution measurements. Several aneuploid nuclei above tetraploid occurred. Nevertheless, with hindsight, an exploration of apoptosis in the primate experiment cited for *P. polonicum* [13] should have been attempted.

In contrast, OTA has a huge literature on toxicity in pigs and poultry, the potent experimental causation of renal cancer in male rats and mice, in vitro experimental toxicology, sophisticated analytical detection in food and feed components, and regulations and legislations for human protection, but there is no proven case of human disease. An experimental diagnosis of renal apoptosis in rats has been noted for OTA according to Haematoxylin and Eosin (H&E) histology [16] and forms a timely basis for comparison here, revealed by specific TUNEL staining, with *P. polonicum*/rat renal histopathology. To avoid any misunderstanding, it should be stated that the *P. polonicum* isolate does not produce OTA.

The present aim has been to illustrate a modern renal histopathological diagnosis of apoptosis in the mycotoxicology of *P. polonicum* in rats after the semi-acute and chronic ingestion of a selective culture extract. Tests for analogous histopathology in response to OTA were also planned. For *P. polonicum*, this study serves to support a prospective report further defining its mycotoxin(s).

## 2. Results

### 2.1. Preliminary Experiments Using P. polonicum-Molded Shredded Wheat

Preliminary studies had explored the importance of rat variety (Fischer, Sprague-Dawley, Lewis, Wistar), gender and age, young adults (~200 g) or weanlings, for expressing the familiar histopathological response to dietary *P. polonicum*. No clear differences were evident except that Sprague-Dawley weanlings in the weight range of 25–40 g expressed the most marked response and were used in some experiments. For continuity with the literature [11] and as a preliminary study, adult male Sprague-Dawley rats were given feed containing either a 20% *P. polonicum*-molded component or a 5% component for 5 days. For the 20% component, toluidine blue-stained sections showed nuclei with condensed and/or fragmented chromatin, i.e., “mitotic figures”, as well as enlarged nuclei, i.e., changes typical of an acute response, in the renal cortico-medullary region where the S3 segment of nephrons is a dominant component. The changes contrasted with the regular histology of a control rat.

A qualitatively similar pathology was observed in rats given the 5% *P. polonicum* feed, but the frequency of lesions was much lower. The outer cortex and medulla in both treatment cases showed no changes. Confirming the well-established features of karyomegaly, necrotic cells, and cells arrested in cell division in the kidneys of rats exposed to a feed with a *P. polonicum*-molded component raised the question of apoptosis as a mechanism of cell death in this context.

The histopathology of rat kidney following 5 days of consuming feed containing a 20% component of shredded wheat molded by *P. polonicum* presents, in the cortico-medullary region, as a complex picture. This involves karyocytomegalic epithelial cells in nephrons, large irregular condensed chromatin bodies that are suggestive of epithelial mitosis and, apparently, necrotic luminal cellular debris in nephrons. The present report is intended to focus on the latter, but this must be set in the context of the other histopathological changes. To establish confidence in the efficient recognition of apoptosis by ApopTag staining, negative controls that were stained only by propidium iodide showed no spontaneous green fluorescence when viewed via the appropriate blue filter. Control rats, given only normal feed, showed only very rare fluorescence in the kidney sections; the observed fluorescence in *P. polonicum*-treated rats was, thus, confidently regarded as indicating apoptosis.

**Figure 1:**
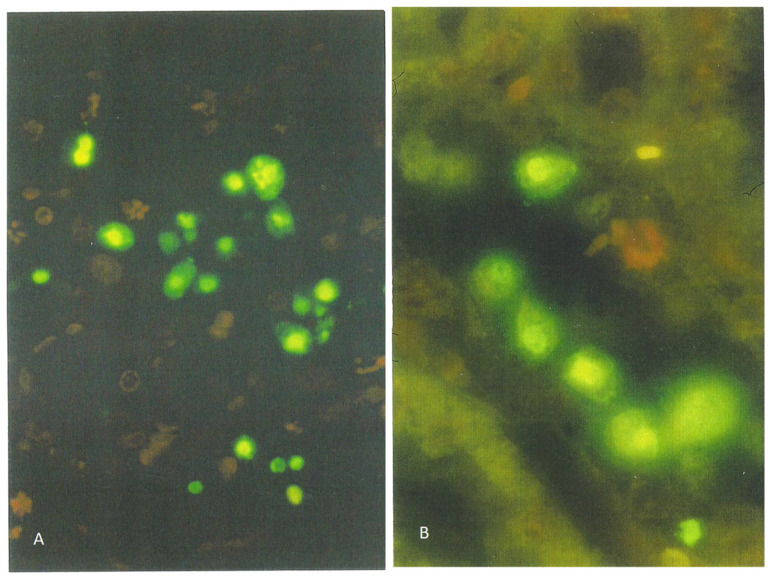
Fluorescein-stained apoptotic nuclei in the renal cortico-medulla of an adult male rat given a 20% *P. polonicum*-molded wheat diet for 5 days ((**A**), ×200). Apoptotic nuclei are depicted in a tubule lumen. A propidium iodide-stained mitotic figure is shown ((**B**), ×450).

Kidney sections of the rat receiving the 20% component were tested for apoptosis using the ApopTag direct labeling kit (Merck Life Sciences, Gillingham, UK), which clearly showed apoptotic nuclei stained the characteristic green-yellow by fluorescein (Figure 1A), in contrast to other nuclei only stained red with propidium iodide. Actually, all the nuclei were stained red by propidium iodide, but the intense green-yellow fluorescence of fluorescein masks the red staining of apoptotic bodies. Apoptotic nuclei, often located toward the lumen of tubules, were confined to the cortico-medullary junction among the many cells with condensed and/or fragmented chromatin. These were often located adjacent to the tubular basement membranes, which were not fluorescein-labeled (Figure 1B).

Further exploratory studies using *P. polonicum* or OTA employed groups of weanling rats (25–50 g).

**Figure 2:**
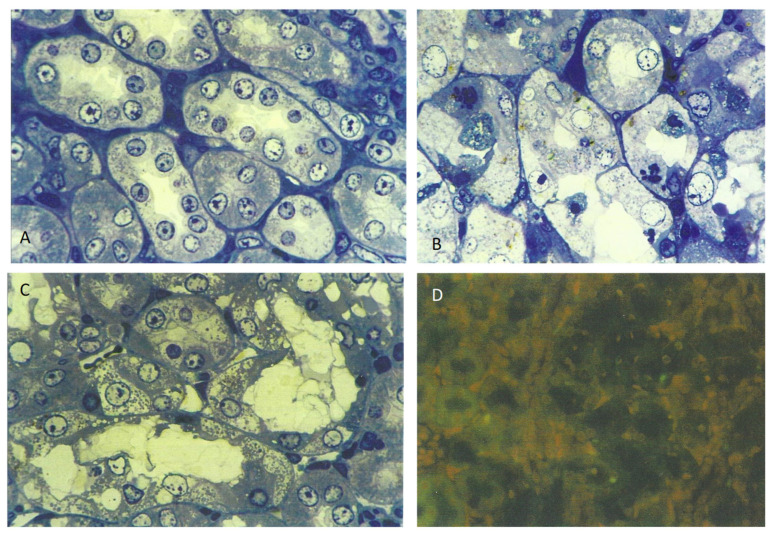
Comparative deviation in weanling rats from normal toluidine blue-stained cortico-medullary histology: (**A**) by 5 days exposure to 20% *P. polonicum*-contaminated feed (**B**) or to ~25 mg OTA/kg b. wt. (**C**), also in the feed. Note the absence of apoptosis, according to ApopTag staining in a weanling rat given OTA (**D**); this may be a consequence of the marked necrotic damage illustrated in (**C**). Settings: (**A**–**C**), 450×; (**D**), 200×.

For OTA, the mycotoxin was given in the feed (0.2 mg or 0.8 mg daily) for 5 days. Histopathological changes in response to the higher OTA dose were also confined to the cortico-medullary junction and involved the extensive loss of nephron epithelia; many cells were necrotic, with eosinophilic cytoplasm, but the changes (stained with toluidine blue, Figure 2C) did not conform to those recorded in response to *P. polonicum* (Figure 2B), both being markedly changed when compared with the control (Figure 2A).

The lower OTA dose elicited only slight changes. The other weanlings given the higher (0.8 mg) OTA in feed were tested for apoptosis using the ApopTag protocol, but none was detected (Figure 2D).

### 2.2. Experiments Using the Cell-Free Extract of P. polonicum/Wheat Fermentation: Recognizing Apoptosis in Both P. Polonicum and OTA Nephrotoxicity after 5 Daily Doses to Groups of 2 or 3 Animals

Seven protocols were applied for administering the 200 g male rat oral intake:

- *P. polonicum* extract from 45 g **or** 15 g shredded wheat substrate in 20 g feed;
- *P.polonicum* extract from 15 g shredded wheat substrate in 0.75 mL water for gavage;
- Ochratoxin A: 1 mg **or** 0.2 mg in bicarbonate (0.3 mL) in 20 g feed **or** for oral gavage, (5 or 1 mg/kg body weight (b. wt.)).

**Figure 3:**
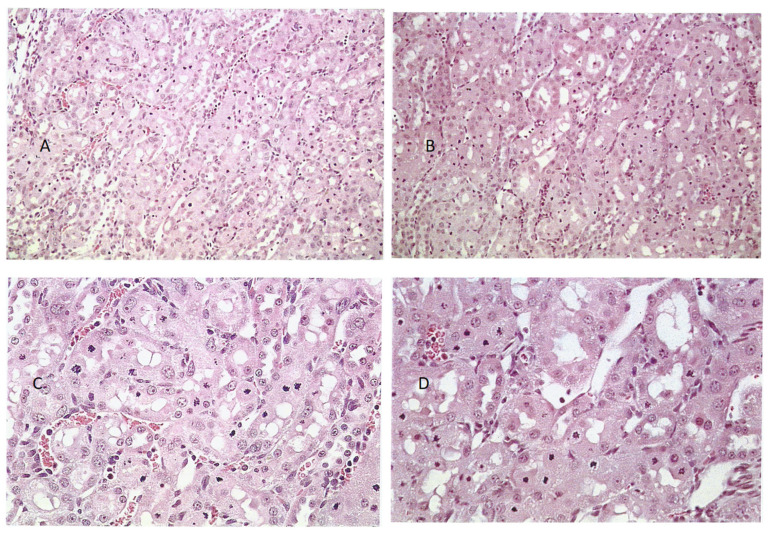
Cortico-medullary histopathology of rats given a feed for 5 days containing extract from 45 g *P. polonicum*-molded wheat Haematoxylin and Eosin (H&E); (**A**) ×100, (**C**) ×200) or 15 g *P. polonicum*-molded wheat (H&E; (**B**) ×100, (**D**) ×200).

**Figure 4:**
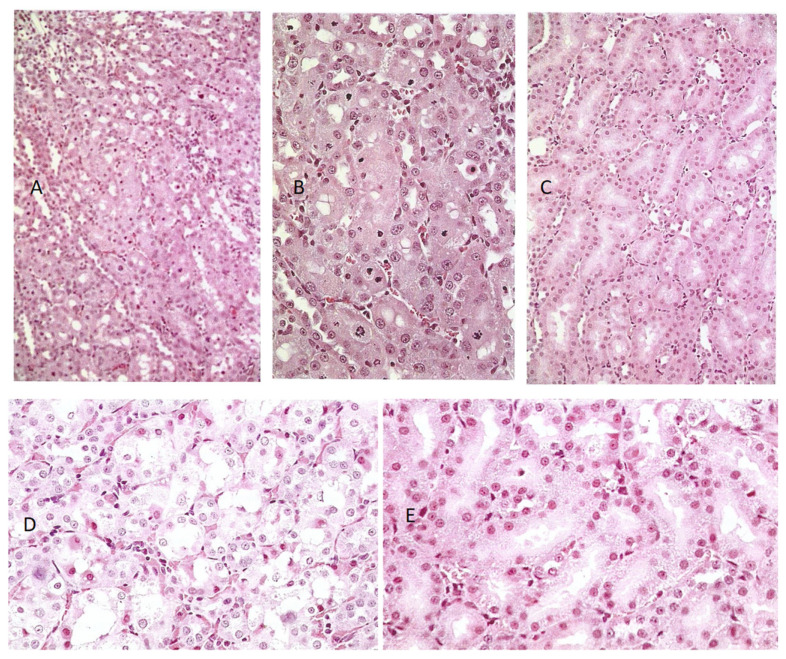
Comparative H&E histopathology in rats given a fermentation extract of 15 g shredded wheat by oral gavage daily for 5 days ((**A**), ×100, (**B**), ×200) with control ((**C**), ×100). Further comparisons show the effect of 1 mg OTA by oral gavage ((**D**), ×200) and in feed ((**E**), ×200).

General renal histopathology, illustrated in hematoxylin and eosin (H&E)-stained sections, showed very frequent necrotic chromatin bodies in the cortico-medullary region of a young adult male (200 g) given an extract from 45 g of shredded wheat molded by *P. polonicum* daily for 5 days, within 20 g of the normal diet (Figure 3A,C). For comparison, the response in another rat given an extract of only 15 g of the molded feed was only slightly less prominent (Figure 3B,D). Where the same extracts were administered once daily by oral gavage, there was still a similar pathology; however, this was clearer at higher magnification (Figure 4A,B), and was set against the normality of a control rat (Figure 4C). Both weight and condition were maintained among these experimental animals.

In rats given OTA, the greatest histopathological change was when 1 mg was given daily by oral gavage; there were frequent necrotic cells and eosinophilic debris within the lumen of tubules in the cortico-medullary region (Figure 4D). In contrast, administration in the feed caused no obvious damage (Figure 4E) and 0.2 mg of the toxin by either route similarly had no effect.

**Figure 5:**
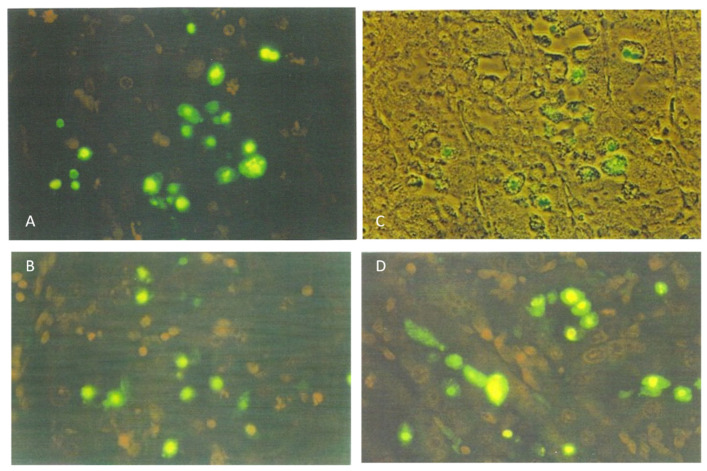
Cortico-medullary region of a rat model, given the higher dose (45 g) of *P. polonicum* fermentation extract in the diet, showing fluorescent apoptotic bodies, most of which either protrude into the tubular lumen or are already free in it (**A**). As seen, also through a phase-contrast blue filter ((**C**), ApopTag ×200). The corresponding illustrations for the lower dose (15 g) given in the feed are shown in (**B**), compared with administering by gavage (**D**). Setting: ×200.

A more impressive response was revealed in the ApopTag-stained preparations, represented by the green-fluorescent bodies in the cortico-medullary region (Figure 5A,C), seen in the young adult rat model that was given an extract from 45 g of shredded wheat molded by *P. polonicum,* given daily for 5 days, within 20 g of the normal diet. This not only verified apoptosis as an aspect of the complex renal histopathological change that can occur in rats consuming feed contaminated by this fungus but also demonstrated that an apoptotic factor could be extracted in alcoholic water. Other rats that were given one-third of the above dose or an extract of the full dose by gavage also showed similar apoptosis (Figure 5B,D) within the cortico-medullary region.

**Figure 6:**
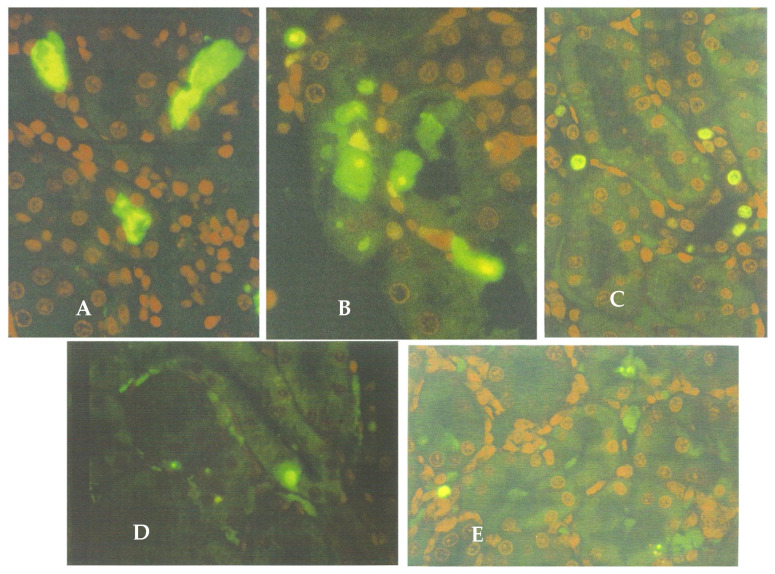
ApopTag staining of the renal cortico-medullary region of rats given OTA daily for 5 days, to show the fluorescent apoptotic bodies (×200). (**A**,**B**) After 1 mg by oral gavage, with marked apoptosis in tubular lumen and epithelia (×200). (**C**) Staining after 0.2 mg by oral gavage, showing discrete apoptosis, particularly in the nephron epithelium (×200). (**D**,**E**) Staining after the administration of 1 mg and 0.2 mg extract, respectively, both in feed, with very occasional apoptosis (×200).

Sprague-Dawley rats (200 g) given the higher OTA dose by oral gavage showed a few fluorescent (apoptotic) bodies oriented within the tubular epithelium, as well as diffuse fluorescence within tubular lumens (Figure 6A,B), possibly representing apoptotic debris. Such histological changes extended not only across the cortico-medullary region but also into both the cortex and medulla. This could logically correlate with the 30 g weight loss sustained during the 5-day dosing period, reflecting a general adverse response to the rather high OTA dose (5 mg/kg b. wt.). In contrast, no weight loss occurred in the other OTA regimens. The lower dose by gavage and the higher dose in feed elicited only 2–3 fluorescent bodies across an entire longitudinal kidney section (Figure 6C,D), and no such fluorescent staining occurred after the lower dose (1 mg/kg b. wt., Figure 6E).

### 2.3. Application of Laser Scanning (Confocal) Microscopy

**Figure 7:**
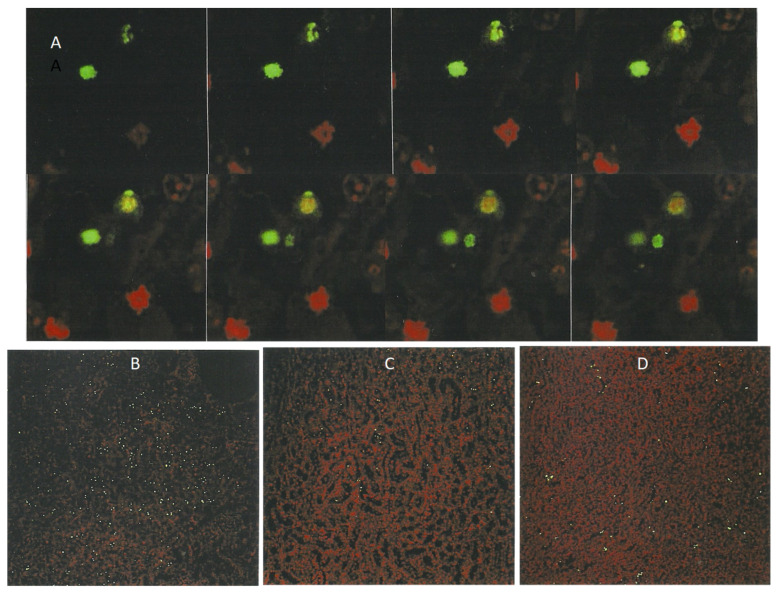
Laser-scanned micrographs of ApopTag preparations for apoptosis caused by *P. polonicum*. A gallery of 8 sequential planes at 0.85 µm intervals through a 6 µm kidney section showing the change of color of certain bodies (labeled with propidium iodide (red) and fluorescein (green)), differentiating between apoptotic (above) and mitotic (below) figures of constituent bodies ((**A**) ×400). Low-magnification (×10) sections in the cortico-medullary region of rats given *P. polonicum* extract in feed at higher (**B**) and one-third lower (**C**) content or OTA at 1 mg/day ((**D**) ×10).

Due to its technical superiority over light fluorescent microscopy, confocal microscopy allowed for the recording of fluorescence in the ApopTag-labeled kidney sections at a much lower magnification, such as ×10 (Figure 7B–D), with a wider view of the renal tissue. This enabled visual proof of the extent of the small fluorescent bodies in the cortico-medullary region, illustrating dose-dependent response relationships among rats given high- or low-dose dietary *P. polonicum* extract; an approximate 140:30 ratio of apoptotic cells is evident. Also illustrated is an analogous response evoked by a rather high regimen for OTA (1 mg by oral gavage); the daily dose approximates to 5 mg/kg b. wt. and approximately 65 apoptotic bodies were revealed in Figure 7D. This illustrates a cortico-medullary background in which the regular arrangement of propidium iodide-stained nuclei showed the normal renal tubule conformation, as in Figure 7B,C. This experiment also verified that the apoptotic *P. polonicum* metabolite is extractable from a fermentation matrix, simply with alcohol and water.

**Figure 8:**
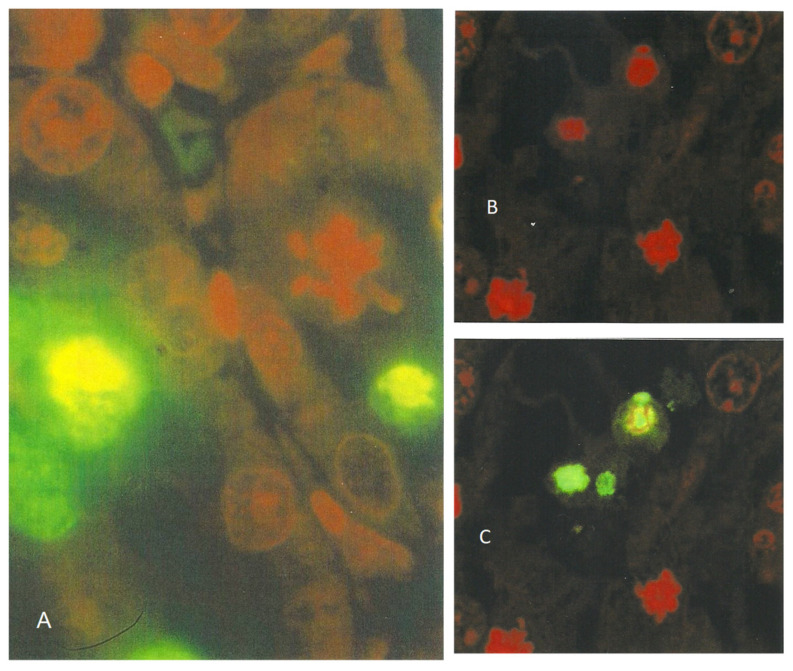
Light-fluorescent micrograph showing bright yellow-green apoptotic bodies and a propidium iodide-stained condensed chromatin resembling a mitotic figure at the kidney cortico-medullary junction of a rat treated with *P. polonicum*. ((**A**) ×450). Laser-scanning micrographs showing apoptotic bodies labeled by propidium iodide only (red, (**B**)) and by fluorescein (green, (**C**)) in ApopTag preparations (×400).

Furthermore, in kidney sections stained with an ApopTag Direct kit, all nuclei are labeled red with propidium iodide, while the apoptotic cells are additionally labeled yellow-green by fluorescein. However, because of the masking effect of fluorescein over propidium iodide, the apoptotic cells are always seen as bright yellow-green-fluorescent bodies under light fluorescence microscopy (Figure 8A). Nevertheless, the powerful laser of the confocal microscope makes it possible to distinguish between the emissions from both fluorescein and propidium iodide within the apoptotic cells (Figure 7A and Figure 8C), allowing differentiation between the genuine cells undergoing apoptosis and those solely carrying green non-specific labeling. Nuclei with condensed chromatin (“mitotic figures”) that are stained only with propidium iodide are also clearly visible in Figure 8.

### 2.4. Intermittent Exposure to P. polonicum for Three Months; Alternating Cycles with Normal Feed

**Figure 9:**
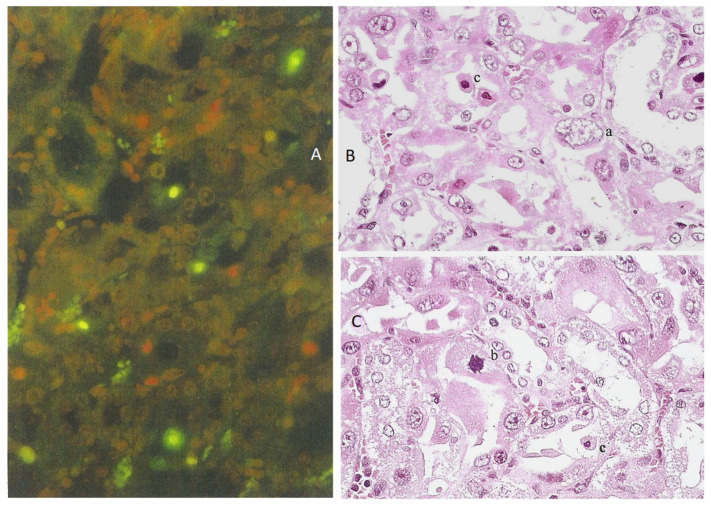
ApopTag-revealed apoptosis after a second 5-day exposure to *P. polonicum* (**A**). Accumulated cortico-medullary histopathological changes, following the fourth cycle of 5-day *P. polonicum* sub-chronic exposure, as various populations of abnormal cells, such as karyocytomegalic ((**B**),a), large mitotic figure ((**C**),b), or pyknotic apoptotic cells ((**B**),c), H&E (×200).

An experiment with four male Sprague-Dawley rats commenced concerning alternating cycles of the standard 5-day 20% *P. polonicum*-molded diet, followed by 3.5 weeks toxin-free, offering opportunities for accumulated histopathological change or partial regression. After the first toxin phase, renal H&E histology in one rat appeared typical of the *P. polonicum* influence already described. The other three rats continued their normal diet for a further 3.5 weeks, at which point two were euthanized for histology. No pyknotic cells or cells with condensed and/or fragmented chromatin that was typical of the first rat’s response to *P. polonicum* were seen. Nevertheless, cells with slightly enlarged nuclei that were mildly karyomegalic occurred in the cortico-medullary region, implying that some nuclear division had occurred during and shortly after the initial *P. polonicum* insult and had persisted. Notably, in kidney sections also subjected to ApopTag staining, a substantial frequency of apoptotic bodies was apparent (Figure 9A).

Experiments with the fourth rat continued for two more cycles, culminating in a fourth 5-day *P. polonicum* treatment, with normal conditions continuing. Kidney H&E histopathology showed a striking response during the three-month experiment, wherein a large proportion of abnormally enlarged cells had much-enlarged nuclei, thus constituting karyocytomegaly. Nephron epithelia with condensed and/or fragmented chromatin were also prominent in the now-distorted cortico-medullary region. Some of these were much larger than those in response to a single 5-day exposure (illustrated in Figure 3). This suggested the cumulative building of specific nuclei during the cycles of *P. polonicum* exposure. Nevertheless, the eosinophilic appearance of the corresponding cytoplasm suggested a concomitant degree of necrosis. Another population of abnormal cells had round or elliptical nuclei in the eosinophilic cytoplasm that protruded into the nephron lumens, extending also into the medulla (Figure 9B,C). These might be apoptotic since they resemble fluorescein-stained bodies in a previous ApopTag-stained example (Figure 9A). It was concluded that intermittent exposure to *P. polonicum* resulted in the induction of prominently karyocytomegalic cells through the cycles of toxic insults, presumably exacerbated by the last one. The persistent evidence takes the form of a combination of “mitotic figures” composed of condensed or fragmented chromatin.

### 2.5. Comparative Continuous-Exposure Responses to Dietary P. polonicum or Ochratoxin A

Male Sprague-Dawley rats (180 g) were given the 20% *P. polonicum*-contaminated diet, two for 3 weeks, one for 2 months, and one for 3 months. An additional rat was given a diet contaminated with OTA (0.4 mg/rat daily: ~2 mg/kg b. wt.), also for 3 months, for comparison. All treatments were well tolerated.

**Figure 10:**
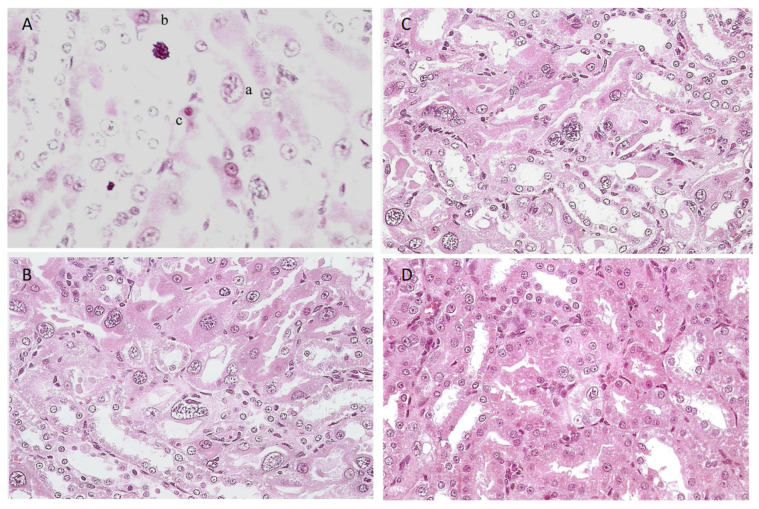
Cortico-medullary histopathology after 3 weeks of dietary exposure to *P. polonicum*-molded feed, showing (**A**) various abnormal cells: karyomegaly (a), large mitotic figure with condensed chromatin (b), and necrotic cells (c). Histopathology after 3 months, showing karyocytomegalic cells and cells with multiple nuclei in tubules, with distorted conformation (**B**,**C**). Comparison with mild karyomegaly within the regular tubular conformation, after 3 months of dietary OTA (0.4 mg daily in feed, (**D**) H&E (×200)).

All rats given *P. polonicum* developed prominent karyocytomegaly, expressed both in the frequency and magnitude of the histopathological change. Additionally, cells with large condensed or fragmented chromatin were seen. A replicated example at 3 weeks is shown in Figure 10A. Nevertheless, these histological abnormalities were most marked in the rat exposed to *P. polonicum* for the longest period, in which the proximal convoluted tubules had lost their arrangement at the cortico-medullary junction and cells with multiple nuclei were evident (Figure 10B,C). The rat that was exposed for two months presented intermediate karyocytomegaly. Although ApopTag staining for the detection of apoptosis had not yet been performed, apoptotic cells were recognized morphologically according to their appearance and location in H&E sections, but they were rare.

In the rat given three months of dietary OTA, only mild karyomegaly occurred within the regular cortico-medullary conformation (Figure 10D), contrasting with the distortions from exposure to *P. polonicum*.

## 3. Discussion

In addition to summarizing the immediate experimental findings on rat renal histopathological responses to the ingestion of *P*. *polonicum* extrolites, along with some comparison with the responses to OTA, the modern evolution of *Penicillium* taxonomy will need to be addressed to highlight some apparent uncertainties in using this taxonomy, particularly for *P. polonicum* and *P. aurantiogriseum* in the recent literature.

Confirmation and refinement of the former general histopathology regarding the dietary exposure of rats to a wheat substrate molded by *P. polonicum*, which is of Balkan origin, has given us an opportunity to focus on the renal pyknotic nuclei and apoptosis and to make some preliminary comparison with that caused by OTA. Finding that progressive renal karyomegaly leading to karyocytomegaly can be evident in both weanling and adult rats, simply via an extract in the feed, enables economy in the bioassays necessary to recognize the elusive toxin(s). Notably, nephrotoxins will remain in an alcohol extract of the *P. polonicum*-molded fermentation of wheat from which, after evaporation, the excess fungal sterol can be precipitated with water. Incorporating the product into powdered feed to mimic the natural human or animal intake also allows the opportunity for evaporation of the traces of residual alcohol before consumption.

Since raising the question concerning any *P. polonicum* contribution by apoptosis to the chronic renal atrophy of BEN [11], this study offers a more comprehensive demonstration that at least most of the pyknotic nuclei in the cortico-medullary nephrons of the H&E-stained kidneys of rats given OTA are apoptotic, according to TUNEL-based histology that confirms apoptosis as contributing at least part of *P. polonicum*’s nephrotoxicity. One subsequent report on apoptosis as a part of OTA nephropathy in the rat model [16] was even more assertive concerning OTA’s putative involvement in the pathogenesis of Balkan endemic nephropathy, offering findings after daily administration over several weeks at an overall rate a little higher than the high dose of the NTP study [17]. Apoptosis was diagnosed after intraperitoneal OTA toxicity was assigned to the small, condensed chromatin bodies deeply stained for such purposes in H&E histology preparations. The extent to which this equates to the specificity of the TUNEL technique is an open question. However, in a further description of the same experiment, H&E staining specifically excluded simple necrosis [18]. The enteral administration of OTA for the present experiments makes direct comparison difficult, but the findings confirm apoptosis for *P. polonicum* as a not-unexpected mycotoxicological attribute. Historically for OTA, the first major rat lifetime exposure study [17] used oral gavage because of its accuracy of dosing in a toxicological context, but predictably slowed bioavailability by delivering the dose in a corn oil vehicle. The present OTA delivery used contrasting oral gavage in an aqueous vehicle or incorporation in the feed for that whole day. The Croatian study’s [16] use of an intraperitoneal route would have given quite quick direct insults to the renal parenchyma. Such administration near the kidneys, although needing only a short circulatory vascular pulse to the renal artery, would of course enable maximum toxicological efficiency, while being non-natural. A single 1 mg/kg dose to female Wistar rats caused a few cortical apoptoses across a kidney section the next day, declining numerically during the following 9 days. After the same dose daily three times per week for 4 weeks, ~100 apoptotic nuclei were recorded across a kidney section, assuming that H&E staining always diagnoses TUNEL histopathology. Presumably, female rats were used because the incidence of endemic nephropathy in Croatia is higher in women [18] and OTA might somehow generate the chronic fibrotic pathology of the Balkan disease.

The recognition of apoptotic nuclei in Wistar male rat kidneys by TUNEL staining after chronic exposure to OTA [19] was also achieved after 2 months of daily gavage in oil at a dose slightly less than that of the mean daily high dose in the NTP study [17], which had caused significant renal cancer much later in life. The exposure-related occasional incidence of karyomegaly and pyknotic nuclei in cortical nephrons in H&E preparations was illustrated, as were fluorescent apoptotic bodies identified specifically by the TUNEL protocol. Another study [20], using an even higher gavage OTA dosage in oil to Sprague-Dawley males for 2 weeks (0.5 mg/kg b. wt.), also caused renal apoptosis, as illustrated by TUNEL staining. However, the serum OTA concentration achieved in the first experimental study was nearly 10 µg/mL which is 10,000 times greater than that measured for a European human exposed to a normal diet; it is important to have a realistic perspective when extrapolating from an experimental rodent to a human model.

In the present study, alternating short periods of dietary *P. polonicum* exposure of young rats with subsequent longer periods of uncontaminated diet over 3 months led to the progressive expansion of karyomegalic nuclei over the 3-month period. However, the findings raise the question of whether the histological picture of progressively increasing karyomegalic ploidy during repetitive exposure to *P. polonicum* is driven within replacement nephron epithelial cells, consequent on replacing the apoptoses that had occurred during the juvenile exposure phase. Continuous *P. polonicum* exposure for 3 months also supported expansive karyocytomegaly; in contrast, continuous dietary OTA exposure at a relatively high dose (2 mg/kg b. wt.) only caused mild karyomegaly within an otherwise undisturbed nephron architecture.

The question arises, therefore, whether local nephron epithelial repair after apoptosis in response to *P. polonicum* nephrotoxicity differs from that in response to OTA, which can easily cause more extensive local cytotoxicity in the cortico-medullary region (Figure 2C). Notably, the DNA ploidy distribution in rat kidneys after 4 weeks of *P. polonicum* dietary exposure [15] caused several aneuploid nuclei in the tetraploid range, but also a few toward octoploid. Such nuclei could be unstable and a potential matter of concern [21]. OTA in male rats is capable of forming renal tumors exhibiting a wide range of unstable aneuploidy [15].

A two-week pathology study [22] with male Sprague-Dawley rats compared dietary *P. polonicum* with oral gavage of the *P. citrinum* mycotoxin, citrinin, which shares its pentaketide structure with a similar moiety of OTA [23]. A 10% *P. polonicum*-molded shredded wheat diet triggered the histopathological changes as presently described, combined with only mild cytotoxicity, all in the same S3 kidney region. Citrinin (2.5 mg/kg) elicited cytotoxicity but caused no nuclear changes when administered alone, but, when combined with the *P. polonicum* regimen, pathological changes were only as those for *P. polonicum.* No significant pathologies in the stomach, small intestine, spleen, thymus gland or lung were associated with the *P. polonicum* regimen. For all regimens, urinary osmolarity decreased, associated with slight glucosuria and an impaired concentration capacity of the kidneys. Urinalysis showed the increased activity of y-glutamyl transpeptidase where rats received *P. polonicum*, also demonstrating the elevated urinary composition of low-molecular-weight proteins. The latter finding predates the proposed role of small serum proteins binding OTA [24], together, salvaged into rat cortical nephrons’ proximal tubule epithelia from the glomerular filtrate. A question now arises concerning whether any analogous mechanism might be operating for a *P. polonicum* nephrotoxic mycotoxin.

**Figure 11:**
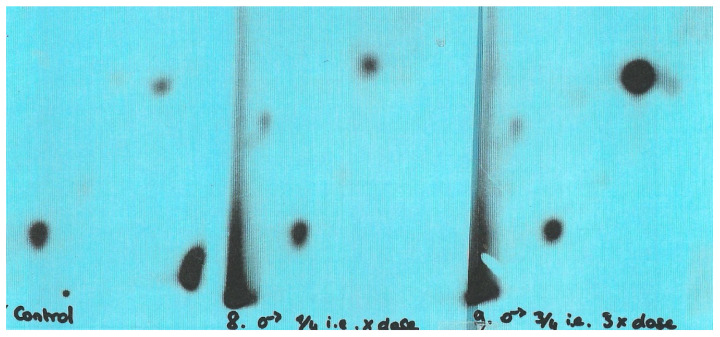
Autoradiographs of polyethyleneimine-cellulose chromatography of ^32^P-post-labeled DNA adducts from rat kidney. Left, control. Center, 5 days’ feeding with a diet containing an extract from 3 g shredded wheat substrate molded for 20 days by *P. polonicum*. The right image is as in the center, but refers to 9 g shredded wheat extract. Principal samples—a specific adduct attributed to *P. polonicum* has measured a ^32^P disintegration ratio of 1:5. A minor spot to the right at a higher dose has a similar ratio with the lower-dose image.

The severity of nephrotoxic responses to environmental Penicillia, whether to OTA from *P. verrucosum* or *P. nordicum*, or to the mycotoxins of *P. polonicum,* has long been conditional not only on the dose magnitude but also on the delivery mode [25]. When there is a marked response, oral gavage gives a greater response than natural delivery in feed; for OTA, this has also been reflected in the mycotoxin’s plasma concentration after repetitive dosing. The principle was extended to apply to the accumulation of DNA adducts in a general exploration of rat kidney DNA in the specialist laboratory of Professor A. Leszkowicz, Toulouse, after a range of exposures to *P. polonicum* [26]. Although most experimental permutations yielded unremarkable findings, the inclusion of *P. polonicum* fermentation extract in the diet caused not only the characteristic histopathological changes already illustrated above but also created one prominent DNA adduct that was dose-related. This was proportionately represented across a threefold difference in dose by a fivefold numerical differential (Figure 11). Therefore, a further aspect of *P. polonicum* nephrotoxicology in the rat model is added, although the amount of the genotoxin in the *P. polonicum* extract consumed over 5 days by adult rats is unknown, as is whether there is any relationship with karyomegaly. For other mammals, the *P. polonicum* histopathology has been seen in guinea pigs and pigs but not in Balb-C mice [27]. Studies in hamsters, contemporary with the rat experiments at Imperial College in the 1980s, showed no histopathological changes after dietary exposure [10].

As is concurrent with the study of its nepropathic potential [5], the former Bulgarian *P. verrucosum* var. *cyclopium* had been found to produce the alkaloid auranthine [28]. Another alkaloid, a benzodiazepine named anacine [29], was later described as a metabolite of a Yugoslavian isolate (IMI 357488), collected in the hyperendemic nephropathy village of Kaniza [7]) and authenticated as *P. aurantiogriseum* Dierckz [6,30]. Subsequently, it is stated [12] that “the original isolate (IMI 180922A) investigated by Barnes et al. [5] as *P. aurantiogriseum* was correctly identified”. It is not clear whether this amplification of the original literature means that it was as recognized before or after its revised taxonomic status [12,31]; the suffix A, added to the simple IMI number as cited [12,30], is also not explained. Ultimately, the culture of IMI 180922 had been supplied directly to P.M. by P. Austwick [5] and revealed nephropathy in rats, enabling the first description of auranthine as a co-metabolite with penicillic acid and verrucosidin [28].

Consequently, the previous discussion is relevant to the recent revision of the auranthine structure [32] since the Bulgarian *P. verrucosum* var. *cyclopium* = *P. aurantiogriseum* = *P. polonicum* nomenclature, spanning over 40 years, implies that further study of auranthine would need to be conducted with a modern *P. polonicum*. Thus, structural revision using a modern, defined *P. aurantiogriseum* isolate (CBS 112021 [32]) could not necessarily be expected to biosynthesize auranthine without access to a reference sample. Unfortunately, none of that sample remains. However, the revised structure, aided by X-ray crystallography, was based on biosynthetic conditions, including a substantial glutamine additive (~15 g/L) to the medium. That additive might reasonably be regarded as not only enriching the nitrogen source but also potentially directing the biosynthesis of a glutamine-derived extrolite. Thus, in perhaps not using the correct fungus, and using a fermentation nutritionally enriched to achieve an increased metabolite yield, the revised structure may indeed widen its occurrence as a *P. aurantiogriseum* metabolite with a weak cytotoxicity profile [32]. Notably, however, a co-metabolite, aurantiamine, markedly decreased the viability of HepG2 cells at 30 µM and above [32]. In our experience, practical differentiation in agar cultures between modern *P. polonicum* and *P. aurantiogriseum* is not easy; it is partly conditioned by the individual perception of color, as was also problematic between the former *P. aurantiogriseum* and *P. commune* in a Croatian study c. 30 years ago [7]—the two were subsequently acknowledged as being synonymous [31]. Notably, *P. commune* isolates, both from Yugoslavia and Bulgaria, and a *P. aurantiogriseum* from Yugoslavia had all had been shown to produce auranthine [7]. While a structural revision after 40 years in the light of new analytical findings is always welcome, it is vital to be sure that the recent revision for auranthine actually relates to the same substance as formerly described [28]. The re-appraisal of auranthine as a structurally characterized metabolite within the *Penicillium* section, *Viridicata* series, *Viridicata* under simple cultural conditions by an authenticated fungus and augmented by biosynthetic evidence would be helpful. Nevertheless, none of the recognized *P. polonicum* extrolites (penicillic acid, verrucosidin, verrucofortines, aspterric acid, anacine, puberulines, cyclopenins [33]) has apparently not yet been tested in terms of the present rat nephropathy.

Notably for the original description of the rat nephropathy described here [5], foodstuff crop samples were collected during the early 1970s in those Balkan areas hyperendemic for the Balkan (endemic) nephropathy. Of three collected from Yugoslavia and Bulgaria and identified as *P. verrucosum var. cyclopium,* only one, from maize in Bulgaria and originally assigned within the *P. cyclopium* series [34] but later cited as *P. verrucosum var. cyclopium* [35], was used for the nephropathy studies in rats [5], although the other two isolates were similarly toxic. Twenty years later, in similar localities [7,8], similar fungi were isolated and identified as *P. aurantiogriseum* and *P. commune*, according to the currently revised *Penicillium* taxonomy [6] (still not yet embellished further by color illustration, although their appearance was subsequently well illustrated [36]). Further taxonomic revision followed [12,31], resolved partly according to the distinctive patterns of secondary metabolites. This revision retained *P. aurantiogriseum* for a more limited application (notably excluding *Penicillia* producing ochratoxin A) and revived *P. polonicum* Zaleski [37] as a distinct entity. The *P. aurantiogriseum* forms coincided with the Balkan isolates that were designated as such [7,8] in the early 1990s, many of which were shown to be nephrotoxic in rats; however, others designated as *P. commune* on account of colony morphology on agar media were also nephrotoxic. At least one *P. aurantiogriseum* or *P. commune* representative from each foodstuff commodity, as studied in Yugoslavia and Bulgaria, demonstrated the karyomegaly pathology in rats as described here, to which a high consistency in expressing nephropathy might actually have occurred. The assignment of this nephropathy as a taxonomic characteristic of both *P. aurantiogriseum* and *P. polonicum,* but not of *P. commune,* was the situation in 2004 [33] but this remains to be re-evaluated.

Diverse examples of recent biochemical publications attributed for *P. polonicum* isolated from different parts of the world and from both terrestrial and marine environments may also be stretching the taxonomic criteria (for example, see [38,39,40]). Caution and mycological rigor are also important in assigning natural isolates to *P. polonicum* [41]. A specific illustration of the terverticillate sporophores of the nephropathic mold highlighted by Barnes et al. [5] is given a decade later [42]. There is clearly a need for well-disseminated genome characterization in assigning fungi to *P*. *polonicum,* bearing in mind that its original description nearly a century ago was from continental Europe (Poland). For reference, cultured material from the present studies, archived privately and probably suitable for genome analysis, is available on request to P.M. An earlier (c. 1989) deposit, then designated *P. commune* from Bulgaria and producing auranthine, is IMI.180922.

The recent notable publication of a Croatian study of fungal contaminants of traditional dry-cured meat products characterized *P. polonicum* and *P. commune* as being among the most abundant and widespread contaminants for which genome analyses were made [43]. However, the authors seemed understandably unaware of such *Penicillia* having a likely similarity to or identity with those also taken from the Croatian village of Kaniza [7] (and the manifest generosity of a dry-cured delicacy there), studied many years ago in London in terms of nephrotoxic molds for rats. Clearly, there remains a basis for mutual interest here.

As a programmed cell death mechanism, apoptosis has been extended to include pyroptosis, which is associated with the body’s response to infection and can be expressed as fragmented DNA; it has recently been applied histologically to the in vivo response to OTA [44]. OTA was administered to male mice intermittently by the intraperitoneal route and has some parallels ([16], although that was not cited in [44]) except for the matter of gender. Some small urinary proteins that have a vital role in sensory behavior in both rats and mice have been suggested, and also as transporters of OTA in the blood [24] while acting as a protein-bound complex augmenting the renal excretion of the mycotoxin. After escaping circulation together through glomerular fenestrations, the natural salvage of some of the proteins in cortical nephrons could also deliver OTA into the S3 epithelia. This could not have been occurring in the Croatian study [16] because the rats were female. In the present dosing of OTA via the natural feed consumption pattern, while a 1 mg/kg b. wt. dose, therefore, predictably delivers a higher relative overall toxic renal insult in male rats than any insult to which human females might have been exposed in the hyperendemic Croatian villages [16], the acute intraperitoneal delivery in the rat model must have resulted in a greater overall surge of OTA in rat renal parenchyma.

The published experimental use of OTA to reveal renal toxicological outcomes often quotes doses that are vastly in excess of any regular human dietary experience. Optimizing the delivery to individual animals, as potential models for revealing toxicology, by parenteral routes may appear more accurate, subject to measuring the delivery of very small amounts. In our experience, administration in feed for sub-clinical effects while satisfying appetite has also allowed reasonable accuracy, as well as coming close to the natural circumstances of the intake of environmental toxins. It would be interesting, in terms of the Li et al. publication [44], to see the renal histopathological changes from the intraperitoneal administration of 100 µg OTA/kg on alternate days, and to perform this for both mouse and rat males.

Therefore, it seems important to rationalize the current heterogeneity of *P. polonicum* and to attribute certain aspects of rat nephrotoxicity of some forms involving ploidy proliferation [14] and the nature of programmed cell death to their respective mycotoxins. Specific nuclear death in the nephron proximal epithelia by what appears to be a rather harmless mold contrasts with the mycotoxin OTA, a product of several other Penicillia, which manifest experimentally as either apoptosis or pyroptosis [44]. It is also important to relate the experimental toxicology protocols to normal routes and quantities of exposure to improve the findings’ relevance to human health. Predicting the application of experimental in vitro toxicology involves the consideration that the toxin under study (e.g., OTA) has been applied directly to naked, cultured human or animal cells. The published dosage for significant toxicity often exceeds those which might ever actually occur naturally in vivo. The natural exposure of toxin per os to a particular animal or human tissue (e.g., kidney) needs first to surmount bioavailability barriers. Potential hepatic biotransformation may then occur before the transfer from vascular circulation to the lumen of nephron tubules. This may involve direct transfer from capillaries to those tubule epithelia having multiple metabolic functions, or, via glomerular escape from blood, gain access to urinary flow. Initial studies on *P. polonicum* [5] clearly showed in vitro toxicity; however, in present studies, this is matched by the quite striking histopathology of induced suicide in rat proximal tubule epithelia and progressive karyocytomegaly, all apparently well-tolerated. In contrast, the renal tubule nuclear suicide induced by OTA occurs within its well-recognized cytopathology in animals. The gentle rat renal apoptosis, from *P. polonicum* in feed, deserves further study.

## 4. Materials and Methods

### 4.1. Renal Tissue Preparation for Histology

Experimental rats in the Imperial College animal facility (21 °C, 12-h light-dark cycle) were caged on sawdust and given diet pellets (rat and mouse diet 1, Special Diet Services, Essex, UK) and water ad lib. Animals given mycotoxin experimentally in their feed were caged individually, on absorbent paper that was changed daily, and were given a powdered diet containing the homogenized experimental material, provided in an aluminum dish. The amount of homogenized feed was adjusted to ensure complete daily consumption during a 24-h period, although normally this was mostly at night, based on animal weight and experience (usually 15–20 g for adults).

Prior to conventional hematoxylin and eosin-stained histology, fresh kidney tissue was fixed in 4% buffered formalin for 24 h. For apoptosis detection, fixation took place for 10 h before the transfer to buffered saline. Fixed tissues were embedded in paraffin wax using automated clinical equipment in the Hedley Atkins Unit at Guy’s Hospital, London, then sectioned (3–4 µm) and mounted on glass slides. For subsequent Apoptag staining, vectabond glass was used.

Renal tissue destined for DNA adduct detection was frozen to −80 °C at autopsy.

### 4.2. Histology for Apoptosis

The Apoptag fluorescein direct in situ apoptosis kit is based on the so-called TdT-mediated dUTP-biotin nick and labeling (TUNEL) assay [45]. It is designed for the staining of histology sections from paraffin-embedded kidney samples. The TUNEL assay is based on the specific binding of deoxynucleotidyl transferase (TdT) to the 3′-OH ends of double-stranded or single-stranded DNA, ensuring the synthesis of a poly-deoxynucleotide polymer. The method allows for the in situ visualization of programmed cell death (apoptosis) at the single-cell level, while preserving the tissue architecture, visualizing not only histologically defined apoptotic cells but also morphologically intact cells in the process of necrosis [46]. In following the manufacturer’s protocol for the direct kit, DNA fragments are directly labeled with chains of fluorescein-labeled nucleotides. Finally, propidium iodide was applied to counter-stain the DNA in all of the cells.

For laser-scanning microscopy, the confocal system and microscope used the manufacturer-supplied software (LSM 510 v1.49.44), running on the Windows NT 4.0 operating system. The λ = 488 nm and λ = 453 nm lines of an argon- and helium-neon-ion laser, respectively, were used for dual excitation. Images were collected using oil-immersion objectives (plan-Neoflua, 40×/1.3; plan Apochrome, 63×/1.4). Emission fluorescence from dual-stained sections was separated with a combination of an FITC-type narrow band-pass filter block (505–530 nm). The images were processed via Photoshop v5.0.

### 4.3. Mycotoxins

For the *P. polonicum* fermentation and extraction of the toxic fraction, a 1 L conical flask in which shredded wheat breakfast cereal (60 g), moistened with 25 mL water, had been fermented by *P. polonicum* (IMI 180922) at 17–19 °C for 2 weeks, with occasional agitation, was treated with 20% ethanol in water (250 mL) overnight. The composition of the extractant was important not only to wet the abundant spores but also to minimize the extraction of their mannitol metabolite. The suspension was filtered (Whatman No 50 paper) and extraction was repeated on the residue. The combined filtrate was evaporated down to a small volume in vacuo, stored at 4 °C overnight for the precipitation of residual mannitol, and centrifuged at 5000× *g* rpm for 15 min. The supernatant was used for the rat experiment as an extract of the *P. polonicum* spoilage of a wheat substrate. Briefly, in terms of the fermentation and transformation of wheat into fungal biomass and metabolites, wheat grain carbohydrate and protein forms the principal substrate for efficient transformation into fungal biomass via partial dissipation, as well as during respiration and the transformation of excess sugars into mannitol. The latter, and the fungal sterols, are the principal soluble products during the subsequent extraction with water and alcohol. Thus, the present cell-free extract is a very small proportion of the original wheat substrate but it will still have a complex composition.

OTA was produced by the shaken solid substrate fermentation of *Aspergillus ochraceus* on shredded wheat for 2 weeks [47], extracted with ethyl acetate, and purified by preparative layer chromatography (Camlab silica gel, 1 mm thick) resolved with toluene/ethyl acetate/formic acid (15:4:1). The blue fluorescent OTA band was excised as a powder and eluted with ethyl acetate. The OTA purity was 98%.

## 5. Conclusions

The focal histological demonstration of renal cortico-medullary nuclear pyknosis as apoptosis has been achieved after only a few days of the dietary exposure of rats to a fermentation extract of *P. polonicum*, which was of central European origin. Apoptotic histopathology is also revealed by the similar administration of the worldwide mycotoxin ochratoxin A, although in a more toxic profile. Thereby, a context is set for the further elucidation of *P. polonicum* extrolites that are also in a fractionated fermentation extract, supported also by a rat bioassay. Longer silent dietary exposure, causing karyocytomegaly in the same renal region by an as-yet obscure toxin, is also illustrated and presents a further challenge for the elucidation of etiology.

## Data Availability

Not applicable.
